# Prenatal, newborn and childhood factors and the timing of puberty in boys and girls

**DOI:** 10.1038/s41390-024-03159-7

**Published:** 2024-04-09

**Authors:** Maria Suutela, Matti Hero, Silja Kosola, Päivi J. Miettinen, Taneli Raivio

**Affiliations:** 1 Pediatric Research Center, New Children’s Hospital, Helsinki University Hospital, Helsinki, Finland; 2 Faculty of Medicine, University of Helsinki, Helsinki, Finland; 3 Research, Development and Innovations, Western Uusimaa Wellbeing Services County, Western Uusimaa, Finland; 4 Stem Cells and Metabolism Research Program, Research Programs Unit, Faculty of Medicine, University of Helsinki, Helsinki, Finland

## Abstract

**Background:**

Our aim was to determine if prenatal factors, gestational age, birth weight and length, and childhood body mass index (BMI) are associated with the timing of puberty.

**Methods:**

Our population-based study comprised 4826 girls and 5112 boys born between 1997 and 2002. Multiple linear regression modeled the relationships between the maternal and child predictors and the age at peak height velocity (PHV).

**Results:**

Maternal smoking throughout pregnancy was associated with earlier age at PHV (−1.8 months in girls, 95%CI = −3.2 to −0.3, *p* = 0.015 and −1.7 months in boys, 95%CI = −3.1 to −0.3, *p* = 0.016). Older gestational age predicted later age at PHV in boys. One SDS increase in birth weight led to 1.7 months later age at PHV in girls (95%CI = 1.2 to 2.2, *p* < 0.001) and 0.8 months in boys (95%CI = 0.2 to 1.3, *p* = 0.005). At the age of 9 years, each increment of BMI by 1 kg/m^2^ was associated with 1.7 months (95%CI = −1.9 to −1.6, *p* < 0.001) and 1.3 months (95%CI = −1.4 to −1.1, *p* < 0.001) earlier age at PHV in girls and boys, respectively.

**Conclusions:**

Fetal exposure to smoking can potentially exert enduring effects on pubertal timing. Birth weight and childhood nutritional status are significant determinants of pubertal timing in both sexes.

**Impact:**

- Maternal smoking was associated with earlier timing of puberty and greater birth weight with later timing of puberty in both girls and boys.
- Most previous studies have focused on girls and used surveys to assess pubertal development, but we studied both sexes and used the same objective measure (age at peak height velocity) for the timing of puberty.
- Our study increases knowledge especially regarding factors associated with the timing of puberty among boys.

## Introduction

The secular trend of earlier timing of puberty.^1,2^ is causing concern regarding later health. Early menarche is associated with insulin resistance,^3^ type 2 diabetes,^4,5^ and hypertension^6^ in adulthood, which may partly be mediated by obesity. Earlier timing of puberty is also linked with different cancers, especially sex steroid-sensitive cancers such as breast, ovarian, and prostate cancer.^7,8^

Previously, prenatal factors such as maternal prepregnancy BMI,^9,10^ exposure to certain chemicals, such as parabens and phenols, during pregnancy^11^ and child birth weight^9,12,13^ were associated with the timing of puberty. Further evidence supports the view that rapid weight gain during the first years of life^13,14^ and higher BMI in childhood^15,16^ are associated with earlier timing of puberty especially in girls. In boys, the association of childhood BMI with the timing of puberty is less clear. Some studies have reported that high BMI is associated with later maturation,^17,18^ other studies found no association^19^ and others reported association of high BMI with earlier maturation^20–24^ although the methods for assessing pubertal timing and size of study population varied. A recent systematic review and meta-analysis assessed evidence on the associations between smoking (prenatal and childhood environmental) and the timing of puberty based on 20 studies of which only 3 included boys.^25^ That meta-analysis inferred that prenatal smoking might advance menarche in girls with some uncertainty, but, in boys, no association between prenatal smoking and puberty was apparent. A Danish questionnaire-based study from 2019 found that maternal smoking during pregnancy might advance puberty in both sexes.^26^ Also socioeconomic status and nutritional conditions are considered to have a role in the timing of puberty.^27^

Many of the studies on the factors affecting the timing of puberty have, however, focused on girls, and used age at menarche as a marker for puberty, although menarche is a late sign of puberty.^28^ In boys, the definition of puberty varies greatly (age at voice break, age at first shaving, early growth of penis). Such self-assessment of pubertal maturation is unreliable, as parents and girls tend to underestimate and boys overestimate their pubertal development.^29^ Therefore, there is still a shortage of comprehensive studies examining how maternal and childhood factors are associated with the timing of puberty, including reliable assessments of pubertal timing in both sexes. Smoking and many other prenatal and childhood factors possibly affecting the timing of puberty could be modifiable.

Our hypothesis is that maternal and child-related factors are predictors for the timing of puberty in both sexes. It has been proposed previously that prenatal factors such as low birth weight and undernutrition are linked to health in adulthood.^30–33^ On the other hand, smoking during pregnancy has been associated with low birth weight.^34,35^ We aim to respond to the gaps in current knowledge that stem from limitations in the methods of assessing pubertal maturation and underpresentation of boys in previous studies. The basis of this population-based study was to investigate the associations of prenatal and newborn factors (gestational age, birth weight and length) and childhood BMI at the age of 6 and 9 years with the timing of puberty in both sexes. The timing of puberty was determined by analyzing growth data that allows equal assessment in both boys and girls.

## Materials and methods

### Study population and design

We collected information on children born between 1997 and 2002 in Finland who attended a comprehensive school in the city of Espoo, the second largest city in Finland. The children’s growth and health data from the visits to child health clinics and school health services were obtained from the integrated patient information systems (Effica®, Tieto Inc.). Growth data included all height and weight measurements from child health clinics and school health care. Data obtained from the newborn register of the Finnish Institute for Health and Welfare included background information of the pregnant mother, labor, and the newborn.

### Informed consent and ethical approval

Since the study is entirely register based, no ethical permission was required according to the Finnish Medical Research Act. The Helsinki University Hospital and the city of Espoo approved the study. Written informed consent from the participants/participants’ legal guardian/next of kin was not required to participate in this study in accordance with the national legislation and the institutional requirements.

### Assessment of pubertal timing

We used age at peak height velocity (PHV) as a marker for pubertal timing and it was determined for 13,180 children who also had newborn register information available. The age at PHV was determined from growth curves using polynomial functions. The process has been described in detail and validated recently.^36^ It is an objective way to determine the timing of puberty retrospectively. In this method, a 7th degree polynomial function was fitted to each child’s growth data and the function was derived to determine the age at PHV, the highest growth rate between 7.5 and 17.5 years of age.

### Information from the newborn register and childhood BMI

**Fig. 1:**
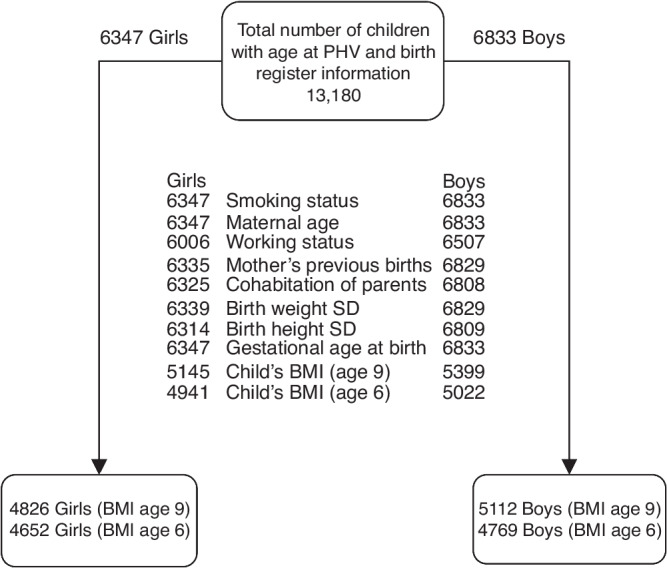
Study outline. The numbers in the middle represent how many of the children had the information of the different birth register variables and BMI. A total of 9938 children, 4826 girls and 5112 boys, had all the variables and BMI at the age of 9 years available and 9421 children, 4652 girls and 4769 boys, all the variables and BMI at the age of 6 years available.

In the newborn register, the smoking status of the pregnant mother was divided into four categories: (i) mother did not smoke, (ii) mother quit smoking during the first trimester (before week 12 + 0), (iii) mother smoked daily also after the first trimester and (iv) smoking status was unknown (2.4%). Other possible maternal factors included the number of previous deliveries, age at delivery, working status (employed or not working (i.e., studying, unemployed, pension, at home with other children)) and cohabitation of parents at the time of delivery/pregnancy. Factors related to the child were gestational age at birth, birth weight standard deviation (SD) score and birth length SD score, and body mass index. We used BMI at the age of 9 years (calculated based on growth assessments for 5145 girls and 5399 boys) and also BMI at the age of 6 years (4941 girls and 5022 boys). For younger ages, growth data was limited. The birth weight SD and birth length SD scores were calculated using Finnish growth reference data for newborns.^37^ After exclusion of children with missing information of all the predictors including BMI at age 9, the final study population included 4826 girls and 5112 boys (Fig. 1). When using BMI at age 6 instead of BMI at age 9 the final study population included 4652 girls and 4769 boys (Fig. 1).

Since most children attend a comprehensive school within their residential area, we used the annual income level of the primary school catchment area where the child attended school in 5th and 6th grade as a proxy for family socioeconomic status. Because the income level of the school catchment area was missing from 893 girls and 780 boys, we conducted a side analysis including this information (3933 girls and 4332 boys).

### Analyses

All statistical analyses were performed with Python programming language (Python 3.7.4). We used multiple linear regression models to find associations between the predictors and age at PHV. The analyses were performed stepwise so that one model included only predictors related to the mother, another model only predictors related to the child and the final model both mother and child-related predictors. The model with both mother (maternal smoking, maternal age, working status, previous births, cohabitation of parents) and child-related (birth weight SDS, birth length SDS, gestational age and childhood BMI) predictors proved to be the best model to describe the associations between the predictors and the age at PHV and was therefore chosen as the final model. We investigated the general trends first with LOWESS (Locally Weighted Scatterplot Smoothing) curves, especially between age at PHV and BMI, and it was close to a linear relationship in both boys and girls. We tested for potential collinearity between the predictors. All the variance inflation factors (VIF) were less than 3 and therefore only low correlations were found. Weak correlation was found between mother’s smoking and birth weight: smoking during pregnancy led to smaller birth weight SD. In addition, children of smoking mothers had higher BMI at age 9 years, and higher birth weight led to higher BMI at age 9. These correlations were minimal and are not considered to interfere with the associations with age at PHV. We also used the Akaike information criteria and analyses on the residuals to test for collinearity. To account for possible selection bias, we compared the ages at PHV of the included and excluded children and found no statistically significant difference between the groups (*p* > 0.05). The excluded children had slightly more smoking mothers than did the included children (1.6 percentage points).

## Results

### Associations of prenatal, newborn and childhood factors with age at PHV

Maternal smoking throughout the pregnancy was associated with earlier timing of puberty in both girls and boys (Table 1). Compared with children of non-smoking mothers, girls reached PHV 1.8 months and boys 1.7 months earlier if their mothers smoked throughout pregnancy. If the mother quit smoking during the first trimester, the age at PHV remained unaffected in both sexes.

**Table 1:** Results from multiple linear regression model with all mother and child-related predictors showing the coefficients, 95% confidence intervals (CI) and *p* values.

	GIRLS (*n* = 4826)			BOYS (*n* = 5112)		
	Age at PHV coef	95% CI	*p* value	Age at PHV coef	95% CI	*p* value
Maternal characteristics						
Maternal smoking						
−throughout pregnancy^a^	**−0.1473**	**−0.266 to −0.029**	**0.015**	**−0.1437**	**−0.261 to −0.027**	**0.016**
−quit in 1. trimester^a^	0.0662	−0.247 to 0.379	0.678	−0.0578	−0.335 to 0.219	0.683
Maternal age	−0.0037	−0.011 to 0.004	0.334	0.0020	−0.006 to 0.010	0.608
Working status	**0.1225**	**0.023 to 0.222**	**0.016**	0.0345	−0.064 to 0.133	0.490
Previous births	−0.0212	−0.052 to 0.010	0.185	0.0169	−0.017 to 0.051	0.327
Cohabitation of parents	0.0302	−0.092 to 0.153	0.629	−0.0080	−0.135 to 0.119	0.902
Child characteristics						
Birth weight SDS	**0.1431**	**0.099 to 0.187**	**0.000**	**0.0634**	**0.019 to 0.108**	**0.005**
Birth length SDS	−0.0423	−0.085 to 0.001	0.053	−0.0031	−0.045 to 0.039	0.885
Gestational age	−0.0025	−0.022 to 0.017	0.806	**0.0271**	**0.008 to 0.046**	**0.004**
BMI at the age of 9	**−0.1428**	**−0.156 to −0.130**	**0.000**	**−0.1053**	**−0.119 to −0.092**	**0.000**
BMI at the age of 6^b^	**−0.1513**	**−0.170 to −0.132**	**0.000**	**−0.1150**	**−0.135 to −0.095**	**0.000**

^a^Compared to non-smokers.

^b^Separate analysis with BMI at the age of 6 years instead of BMI at the age of 9 years, 4652 girls and 4769 boys.

^a^Compared to non-smokers.

The *p* values that are significant are in bold.

The birth weight SDS was positively associated with age at PHV (Table 1). One SD increase in birth weight SD score was associated with 1.7 months later age at PHV in girls and 0.8 months later age in boys. However, when investigating the residuals, most of the variation in birth weight in boys was probably due to the effect of smoking on the age at PHV.

In both sexes, most of the variance of age at PHV was explained by the child’s BMI. Increase in BMI at the age of 9 years by 1 kg/m^2^, led to 1.7 months earlier age at PHV in girls and 1.3 months earlier age at PHV in boys (Table 1). Similar results were found with BMI at the age of 6 in both girls and boys: a BMI increase by 1 kg/m^2^ led to 1.8 months and 1.4 months earlier age at PHV, respectively. Higher BMI was associated with earlier age at PHV also among overweight and obese children. If BMI at the age of 9 years was >90th percentile, a BMI increase by 1 kg/m^2^ in girls led to 1.6 months and in boys to a 1.1 months earlier age at PHV. The corresponding analyses for BMI at the age of 6 years led to a 2.2 months and a 1.2 months earlier age at PHV.

In addition to child BMI, maternal smoking, and child birth weight, in girls, also maternal employment correlated with the age at PHV leading to a 1.5 months later age at PHV compared to those whose mothers were not working. In boys, each additional gestational week was associated with 0.3 months later puberty. Maternal age, number of previous births, cohabitation of the parents and birth length SD score showed no association with the timing of puberty in either sex.

When the annual income level of the school catchment area was added to the analysis, other results remained similar, but in girls, maternal working status became non-significant. There was no relevant association between annual income level and age at PHV.

### Characteristics based on smoking status

The characteristics of the smoking and non-smoking mothers are shown in Table 2. Mothers who continued smoking after the 1st trimester had more children, were younger and were more often out of workforce and less frequently cohabited with the other parent compared to the non-smoking mothers. The children of the smoking mothers were smaller at gestational age and heavier at the age of 9. If the mother quit smoking in the first trimester birth size was unaffected, but the children had gained more weight by the age of 9.

**Table 2:** Characteristics (mother and child-related) when mother either smoked throughout the pregnancy, quit smoking in the 1st trimester or was non-smoking.

	Smoking continued after 1st trimester *n* = 862	Smoking ended in 1st trimester *n* = 120	Non-smoking *n* = 8716	All^b^ *n* = 9938
Previous childbirths mean (SD)	1.02 (1.23)*	0.48 (0.76)*	0.84 (1.06)	0.86 (1.08)
Maternal age mean (SD)	29.34 (5.64)*	29.55 (5.70)*	31.59 (4.70)	31.37 (4.85)
Employed	78.77%*	79.17%*	87.05%	86.11%
Not working^a^	21.23%*	20.83%*	12.95%	13.88%
Cohabitation of parents	68.91%*	69.17%*	89.65%	87.54%
Gestational weeks	39.74 (1.64)	40.00 (1.49)	39.76 (1.68)	39.76 (1.67)
Birth weight SDS	−0.38 (1.03)*	−0.23 (0.97)	−0.04 (1.05)	−0.08 (1.05)
Birth length SDS	−0.32 (1.15)*	−0.04 (1.02)	0.09 (1.06)	0.05 (1.08)
Child’s BMI at age 9	17.74 (2.96)*	17.82 (3.14)*	16.97 (2.37)	17.04 (2.45)
age at PHV (SD)	girls: 11.15 (1.19)* boys: 13.32 (1.17)*	girls: 11.35 (1.30) boys: 13.42 (1.24)	girls: 11.45 (1.18) boys: 13.57 (1.18)	girls: 11.42 (1.18) boys: 13.54 (1.19)

^*^p < 0.05 when compared to non-smoking mothers.

^a^Studying, unemployed, pension, at home with other children.

^b^Including also mothers whose smoking status was unknown.

## Discussion

In this study, we examined the relationship of prenatal factors, birth size, and childhood BMI with the timing of puberty in a comprehensive population-based cohort among both girls and boys. From the predictors used, childhood BMI showed the strongest and negative association with the age at PHV. Moreover, maternal smoking during pregnancy was associated with earlier timing of puberty in both sexes but only if smoking continued throughout the pregnancy. Birth weight SD score had a positive association with the age at PHV.

Earlier age at puberty onset in both girls and boys is a global phenomenon.^1,2,19,38^ This secular trend may challenge the definition of what is considered normal, may concern parents, and may cause burden on the health care system because more children are referred due to the suspicion of precocious puberty.^39–41^ Although the etiology of earlier timing of puberty remains largely unknown, early puberty is linked to a multitude of adverse health outcomes, such as type 2 diabetes and sex-steroid sensitive cancers.^4,7^

Smoking throughout the pregnancy was associated with earlier timing of puberty in both sexes. Maternal smoking during pregnancy has been associated with early^42,43^ or late^44^ menarche, and some studies^45^ report no association at all. A recent meta-analysis concluded that maternal smoking during pregnancy might indeed lower age at menarche for girls, but among boys, an unequivocal puberty-promoting association was lacking.^25^ A Danish study, which accounted for many confounding factors such as prepregnancy BMI, maternal age at delivery, and social class of parents, concluded that maternal smoking during pregnancy might advance puberty in both boys and girls.^26^ This is in agreement with our results, though we found no association between maternal smoking and age at PHV if the mother quit smoking during the first trimester. Other studies also indicate that quitting smoking during the first trimester is beneficial for newborn birth weight.^46,47^ In regard to tobacco smoke exposure during childhood, one Finnish study recently reported that such exposure at home predicts earlier thelarche.^9^ Finally, maternal smoking during pregnancy is associated in adulthood with reduced semen quality and testis size, suggesting a long-lasting effect on boys’ reproductive system.^48,49^

Birth weight was associated with the age at PHV in both sexes. Previous studies on girls have found similar associations between low birth weight and early menarche.^43,45,50,51^ In boys, the results are more ambiguous. In a study by Wohlfahrt-Veje et al, larger birth weight in boys predicted earlier age for attaining testicular volume greater than 3 ml, while predicting later age for Tanner genital stage 4 (G4) or pubic hair stage 4 (P4).^12^ A recent study on preterm boys reported a negative correlation between the degree of prematurity and birth weight and circulating sex steroid levels at age 10, which suggests that, in children with lower birth weight, the hypothalamus–pituitary–testicular axis might be activated earlier.^52^ In our study, however, most of the variation in the birth weight of boys was explained by maternal smoking and thus the effect of birth weight on the age of PHV was quite small.

The strongest predictor of earlier age at PHV was child BMI at the age of 6 and 9 years. This was expected, based on similar results, especially in girls.^15,53–57^ In boys, however, the situation is less clear. For example, Bygdell et al and Oehme et al suggested that the relationship between nutritional status and the timing of puberty did not apply to overweight boys,^58,59^ whereas other investigators have reported that higher BMI is associated with earlier timing of puberty, irrespective of boys’ BMI.^15,60–63^ In our study, BMI at age 6 and 9 years was associated with earlier age at PHV also among overweight boys (BMI>90th percentile or BMI>95th percentile). The World Health Organization defines normal BMI for girls as 12.7 kg/m^2^ to 17 kg/m^2^ at age 6 and 13.1 kg/m^2^ to 18.3 kg/m^2^ at age 9 and for boys a respective 13 kg/m^2^ to 16.8 kg/m^2^ and 13.5 kg/m^2^ to 17.9 kg/m^2^.^64^ The association between child weight and timing of puberty lends credence to the concept that prevention of childhood obesity may modify the timing of puberty in both girls and boys.^62^ In our study already BMI at the age of 6 years, which is well before the onset of puberty, was associated with the timing of puberty.

The strengths of this study include its population-based setting, large study cohort, and objective assessment of the timing of puberty from growth charts instead of surveys. Unfortunately, quantitative and comprehensive data on maternal smoking during pregnancy, and the child’s possible exposure to tobacco smoke at home were unavailable. Other important variables which we had no access to included maternal weight/BMI, lifestyle, gestational diabetes, and family’s income level. Additional lifestyle and socioeconomic factors like these may also contribute to the associations described between maternal smoking during pregnancy and timing of puberty.

In summary, the timing of puberty in both sexes is significantly influenced by maternal smoking during pregnancy, child birth weight, and BMI at ages 6 and 9. This underscores the importance of recognizing smoking and childhood obesity as risk factors for long-term health.

## Data Availability

The data that support the findings of this study are available from Helsinki University Hospital, the city of Espoo and the Finnish Institute for Health and Welfare but restrictions apply to the availability of these data, which were used under license for the current study, and so are not publicly available. Data are however available from the authors upon reasonable request and with permission of Helsinki University Hospital, the city of Espoo and the Finnish Institute for Health and Welfare. Requests to access the datasets should be directed to the corresponding author.

## References

[1] Eckert-Lind, C. et al. Worldwide secular trends in age at pubertal onset assessed by breast development among girls: a systematic review and meta-analysis. *JAMA Pediatr.* **174**, e195881 (2020). 32040143 10.1001/jamapediatrics.2019.5881 PMC7042934

[2] Ohlsson, C. et al. Secular trends in pubertal growth acceleration in Swedish boys born from 1947 to 1996. *JAMA Pediatr.* **173**, 860–865 (2019). 31329245 10.1001/jamapediatrics.2019.2315 PMC6647355

[3] Zhang, Z., Hu, X., Yang, C. & Chen, X. Early age at menarche is associated with insulin resistance: a systemic review and meta-analysis. *Postgrad. Med.* **131**, 144–150 (2019). 30560708 10.1080/00325481.2019.1559429

[4] Cheng, T. S., Day, F. R., Lakshman, R. & Ong, K. K. Association of puberty timing with type 2 diabetes: a systematic review and meta-analysis. *PLoS Med* **17**, e1003017 (2020). 31905226 10.1371/journal.pmed.1003017 PMC6944335

[5] Ohlsson, C., Bygdell, M., Nethander, M. & Kindblom, J. M. Early puberty and risk for type 2 diabetes in men. *Diabetologia* **63**, 1141–1150 (2020). 32201902 10.1007/s00125-020-05121-8 PMC7228987

[6] Bubach, S. et al. Early menarche and blood pressure in adulthood: systematic review and meta-analysis. *J. Public Health* **40**, 476–484 (2018). 10.1093/pubmed/fdx118 28977577

[7] Day, F. R. et al. Genomic analyses identify hundreds of variants associated with age at menarche and support a role for puberty timing in cancer risk. *Nat. Genet.* **49**, 834–841 (2017). 28436984 10.1038/ng.3841 PMC5841952

[8] Al-Ajmi, K., Lophatananon, A., Ollier, W. & Muir, K. R. Risk of breast cancer in the UK biobank female cohort and its relationship to anthropometric and reproductive factors. *PLOS One* **13**, e0201097 (2018). 30048498 10.1371/journal.pone.0201097 PMC6062099

[9] Savinainen, S. E., Viitasalo, A., Sallinen, T. M., Jääskeläinen, J. E. S. & Lakka, T. A. Child-related and parental predictors for thelarche in a general population of girls: the PANIC study. *Pediatr. Res.* **88**, 676–680 (2020). 32050255 10.1038/s41390-020-0802-0

[10] Zhou, J. et al. Maternal pre-pregnancy body mass index, gestational weight gain, and pubertal timing in daughters: a systematic review and meta-analysis of cohort studies. *Obes. Rev.* **23**, e13418 (2022). 35014751 10.1111/obr.13418

[11] Harley, K. G. et al. Association of phthalates, parabens and phenols found in personal care products with pubertal timing in girls and boys. *Hum. Reprod.* **34**, 109–117 (2019). 30517665 10.1093/humrep/dey337 PMC6295961

[12] Wohlfahrt-Veje, C. et al. Pubarche and gonadarche onset and progression are differently associated with birth weight and infancy growth patterns. *J. Endocr. Soc.* **5**, bvab108 (2021). 34250379 10.1210/jendso/bvab108 PMC8262798

[13] Karaolis-Danckert, N., Buyken, A. E., Sonntag, A. & Kroke, A. Birth and early life influences on the timing of puberty onset: results from the DONALD (DOrtmund Nutritional and Anthropometric Longitudinally Designed) Study. *Am. J. Clin. Nutr.* **90**, 1559–1565 (2009). 19828713 10.3945/ajcn.2009.28259

[14] Choe, Y. et al. Rapid weight gain in early life is associated with central precocious puberty in girls, not in boys - a nationwide population-based study in Korea. *Front. Endocrinol.* **14**, 1210995 (2023). 10.3389/fendo.2023.1210995 PMC10381025 37522114

[15] Liu, G. et al. Obesity is a risk factor for central precocious puberty: a case-control study. *BMC Pediatr.* **21**, 509 (2021). 34784914 10.1186/s12887-021-02936-1 PMC8594221

[16] Reinehr, T. & Roth, C. L. Is there a causal relationship between obesity and puberty? *Lancet Child Adolesc. Health* **3**, 44–54 (2019). 30446301 10.1016/S2352-4642(18)30306-7

[17] Lee, J. M. et al. Body mass index and timing of pubertal initiation in boys. *Arch. Pediatr. Adolesc. Med.* **164**, 139–144 (2010). 20124142 10.1001/archpediatrics.2009.258 PMC4172573

[18] Wang, Y. Is obesity associated with early sexual maturation? A comparison of the association in American boys versus girls. *Pediatrics* **110**, 903–910 (2002). 12415028 10.1542/peds.110.5.903

[19] Karpati, A. M., Rubin, C. H., Kieszak, S. M., Marcus, M. & Troiano, R. P. Stature and pubertal stage assessment in American boys: the 1988-1994 Third National Health and Nutrition Examination Survey. *J. Adolesc. Health* **30**, 205–212 (2002). 11869928 10.1016/s1054-139x(01)00320-2

[20] Juul, A., Magnusdottir, S., Scheike, T., Prytz, S. & Skakkebaek, N. E. Age at voice break in Danish boys: effects of pre-pubertal body mass index and secular trend. *Int. J. Androl.* **30**, 537–542 (2007). 17459124 10.1111/j.1365-2605.2007.00751.x

[21] Buyken, A. E., Karaolis-Danckert, N. & Remer, T. Association of prepubertal body composition in healthy girls and boys with the timing of early and late pubertal markers. *Am. J. Clin. Nutr.* **89**, 221–230 (2009). 19056586 10.3945/ajcn.2008.26733

[22] Sandhu, J., Ben-Shlomo, Y., Cole, T. J., Holly, J. & Davey Smith, G. The impact of childhood body mass index on timing of puberty, adult stature and obesity: a follow-up study based on adolescent anthropometry recorded at Christ’s Hospital (1936-1964). *Int. J. Obes.* **30**, 14–22 (2006). 10.1038/sj.ijo.0803156 16344844

[23] He, Q. & Karlberg, J. Bmi in childhood and its association with height gain, timing of puberty, and final height. *Pediatr. Res.* **49**, 244–251 (2001). 11158521 10.1203/00006450-200102000-00019

[24] Brix, N. et al. Childhood overweight and obesity and timing of puberty in boys and girls: cohort and sibling-matched analyses. *Int. J. Epidemiol.* **49**, 834–844 (2020). 32372073 10.1093/ije/dyaa056 PMC7394964

[25] Chen, Y. et al. Association of prenatal and childhood environment smoking exposure with puberty timing: a systematic review and meta-analysis. *Environ. Health Prev. Med.* **23**, 33 (2018). 30021511 10.1186/s12199-018-0722-3 PMC6052528

[26] Brix, N. et al. Maternal smoking during pregnancy and timing of puberty in sons and daughters: a population-based cohort study. *Am. J. Epidemiol.* **188**, 47–56 (2019). 30239589 10.1093/aje/kwy206 PMC6321801

[27] Parent, A.-S. et al. The timing of normal puberty and the age limits of sexual precocity: variations around the world, secular trends, and changes after migration. *Endocr. Rev.* **24**, 668–693 (2003). 14570750 10.1210/er.2002-0019

[28] Brix, N. et al. Timing of puberty in boys and girls: a population‐based study. *Paediatr. Perinat. Epidemiol.* **33**, 70–78 (2019). 30307620 10.1111/ppe.12507 PMC6378593

[29] Rasmussen, A. R. et al. Validity of self-assessment of pubertal maturation. *Pediatrics* **135**, 86–93 (2015). 25535262 10.1542/peds.2014-0793

[30] Barker, D. J. Fetal origins of coronary heart disease. *BMJ* **311**, 171–174 (1995). 7613432 10.1136/bmj.311.6998.171 PMC2550226

[31] Barker, D. J. et al. Fetal nutrition and cardiovascular disease in adult life. *Lancet* **341**, 938–941 (1993). 8096277 10.1016/0140-6736(93)91224-a

[32] Barker, D. J., Winter, P. D., Osmond, C., Margetts, B. & Simmonds, S. J. Weight in infancy and death from ischaemic heart disease. *Lancet* **2**, 577–580 (1989). 2570282 10.1016/s0140-6736(89)90710-1

[33] Hales, C. N. & Barker, D. J. Type 2 (non-insulin-dependent) diabetes mellitus: the thrifty phenotype hypothesis. *Diabetologia* **35**, 595–601 (1992). 1644236 10.1007/BF00400248

[34] Pereira, P. P. et al. Maternal active smoking during pregnancy and low birth weight in the Americas: a systematic review and meta-analysis. *Nicotine Tob. Res* **19**, 497–505 (2017). 28403455 10.1093/ntr/ntw228

[35] Di, H.-K. et al. Maternal smoking status during pregnancy and low birth weight in offspring: systematic review and meta-analysis of 55 cohort studies published from 1986 to 2020. *World J. Pediatr.* **18**, 176–185 (2022). 35089538 10.1007/s12519-021-00501-5

[36] Suutela, M. et al. Timing of puberty and school performance: a population-based study. *Front. Endocrinol.* **13**, 936005 (2022). 10.3389/fendo.2022.936005 PMC9388756 35992102

[37] Sankilampi, U., Hannila, M.-L., Saari, A., Gissler, M. & Dunkel, L. New population-based references for birth weight, length, and head circumference in singletons and twins from 23 to 43 gestation weeks. *Ann. Med.* **45**, 446–454 (2013). 23768051 10.3109/07853890.2013.803739

[38] Aksglaede, L., Sørensen, K., Petersen, J. H., Skakkebaek, N. E. & Juul, A. Recent decline in age at breast development: the Copenhagen Puberty Study. *Pediatrics* **123**, e932–e939 (2009). 19403485 10.1542/peds.2008-2491

[39] Mogensen, S. S. et al. Diagnostic work-up of 449 consecutive girls who were referred to be evaluated for precocious puberty. *J. Clin. Endocrinol. Metab.* **96**, 1393–1401 (2011). 21346077 10.1210/jc.2010-2745

[40] Bräuner, E. V. et al. Trends in the incidence of central precocious puberty and normal variant puberty among children in Denmark, 1998 to 2017. *JAMA Netw. Open* **3**, e2015665 (2020). 33044548 10.1001/jamanetworkopen.2020.15665 PMC7550972

[41] Kang, S., Park, M. J., Kim, J. M., Yuk, J.-S. & Kim, S.-H. Ongoing increasing trends in central precocious puberty incidence among Korean boys and girls from 2008 to 2020. *PLoS One* **18**, e0283510 (2023). 36947549 10.1371/journal.pone.0283510 PMC10032490

[42] Ernst, A. et al. Maternal smoking during pregnancy and reproductive health of daughters: a follow-up study spanning two decades. *Hum. Reprod.* **27**, 3593–3600 (2012). 23034153 10.1093/humrep/des337

[43] Behie, A. M. & O’Donnell, M. H. Prenatal smoking and age at menarche: influence of the prenatal environment on the timing of puberty. *Hum. Reprod.* **30**, 957–962 (2015). 25740885 10.1093/humrep/dev033

[44] Ferris, J. S., Flom, J. D., Tehranifar, P., Mayne, S. T. & Terry, M. B. Prenatal and childhood environmental tobacco smoke exposure and age at menarche. *Paediatr. Perinat. Epidemiol.* **24**, 515–523 (2010). 20955229 10.1111/j.1365-3016.2010.01154.x PMC3070941

[45] Dossus, L. et al. Determinants of age at menarche and time to menstrual cycle regularity in the French E3N cohort. *Ann. Epidemiol.* **22**, 723–730 (2012). 22902044 10.1016/j.annepidem.2012.07.007

[46] Abraham, M. et al. A systematic review of maternal smoking during pregnancy and fetal measurements with meta-analysis. *PLoS One* **12**, e0170946 (2017). 28231292 10.1371/journal.pone.0170946 PMC5322900

[47] Prabhu, N. et al. First trimester maternal tobacco smoking habits and fetal growth. *Thorax* **65**, 235–240 (2010). 20335293 10.1136/thx.2009.123232

[48] Jensen, T. K. et al. Association of in utero exposure to maternal smoking with reduced semen quality and testis size in adulthood: a cross-sectional study of 1770 young men from the general population in five European countries. *Am. J. Epidemiol.* **159**, 49–58 (2004). 14693659 10.1093/aje/kwh002

[49] Axelsson, J. et al. The impact of paternal and maternal smoking on semen quality of adolescent men. *PLoS One* **8**, e66766 (2013). 23840528 10.1371/journal.pone.0066766 PMC3694111

[50] Juul, F., Chang, V. W., Brar, P. & Parekh, N. Birth weight, early life weight gain and age at menarche: a systematic review of longitudinal studies. *Obes. Rev.* **18**, 1272–1288 (2017). 28872224 10.1111/obr.12587

[51] D’Aloisio, A. A., DeRoo, L. A., Baird, D. D., Weinberg, C. R. & Sandler, D. P. Prenatal and infant exposures and age at menarche. *Epidemiology* **24**, 277–284 (2013). 23348069 10.1097/EDE.0b013e31828062b7 PMC3563843

[52] Kvernebo Sunnergren, K. et al. Pre- and peripubertal sex steroids are inversely associated with birth weight in preterm boys. *Clin. Endocrinol.* **98**, 342–350 (2023). 10.1111/cen.14821 36071648

[53] Kaplowitz, P. B., Slora, E. J., Wasserman, R. C., Pedlow, S. E. & Herman-Giddens, M. E. Earlier onset of puberty in girls: relation to increased body mass index and race. *Pediatrics* **108**, 347–353 (2001). 11483799 10.1542/peds.108.2.347

[54] Kaplowitz, P. B. Link between body fat and the timing of puberty. *Pediatrics* **121**, S208–S217 (2008). 18245513 10.1542/peds.2007-1813F

[55] Sørensen, K., Aksglaede, L., Petersen, J. H. & Juul, A. Recent changes in pubertal timing in healthy Danish boys: associations with body mass index. *J. Clin. Endocrinol. Metab.* **95**, 263–270 (2010). 19926714 10.1210/jc.2009-1478

[56] Silventoinen, K., Jelenkovic, A., Palviainen, T., Dunkel, L. & Kaprio, J. The association between puberty timing and body mass index in a longitudinal setting: the contribution of genetic factors. *Behav. Genet.* **52**, 186–194 (2022). 35381915 10.1007/s10519-022-10100-3 PMC9135891

[57] Busch, A. S. et al. Voice break in boys-temporal relations with other pubertal milestones and likely causal effects of BMI. *Hum. Reprod.* **34**, 1514–1522 (2019). 31348498 10.1093/humrep/dez118 PMC6688887

[58] Bygdell, M., Kindblom, J. M., Celind, J., Nethander, M. & Ohlsson, C. Childhood BMI is inversely associated with pubertal timing in normal-weight but not overweight boys. *Am. J. Clin. Nutr.* **108**, 1259–1263 (2018). 30321255 10.1093/ajcn/nqy201 PMC6300589

[59] Oehme, N. H. B. et al. Low BMI, but not high BMI, influences the timing of puberty in boys. *Andrology* **9**, 837–845 (2021). 33544961 10.1111/andr.12985

[60] Ribeiro, J., Santos, P., Duarte, J. & Mota, J. Association between overweight and early sexual maturation in Portuguese boys and girls. *Ann. Hum. Biol.* **33**, 55–63 (2006). 16500811 10.1080/00207390500434135

[61] Lee, J. M. et al. Timing of puberty in overweight versus obese boys. *Pediatrics* **137**, e20150164 (2016). 26817933 10.1542/peds.2015-0164

[62] Aghaee, S. et al. Associations between childhood obesity and pubertal timing stratified by sex and race/ethnicity. *Am. J. Epidemiol.* **191**, 2026–2036 (2022). 35998084 10.1093/aje/kwac148 PMC10144668

[63] Aksglaede, L., Juul, A., Olsen, L. W. & Sørensen, T. I. A. Age at puberty and the emerging obesity epidemic. *PLoS One* **4**, e8450 (2009). 20041184 10.1371/journal.pone.0008450 PMC2793517

[64] Growth reference 5–19 years - BMI-for-age (5–19 years). https://www.who.int/tools/growth-reference-data-for-5to19-years/indicators/bmi-for-age.

